# A highly efficient degradation mechanism of methyl orange using Fe-based metallic glass powders

**DOI:** 10.1038/srep21947

**Published:** 2016-02-23

**Authors:** Shenghui Xie, Ping Huang, Jamie J. Kruzic, Xierong Zeng, Haixia Qian

**Affiliations:** 1 College of Materials Science and Engineering, Shenzhen University, Shenzhen Engineering Laboratory for Advanced Technology of Ceramics and Shenzhen Key Laboratory of Special Functional Materials, ShenZhen, 518060, China; 2 Materials Science, School of Mechanical, Industrial, and Manufacturing Engineering, Oregon State University, Corvallis, OR 97331, USA

## Abstract

A new Fe-based metallic glass with composition Fe_76_B_12_Si_9_Y_3_ (at. %) is found to have extraordinary degradation efficiency towards methyl orange (MO, C_14_H_14_N_3_SO_3_) in strong acidic and near neutral environments compared to crystalline zero-valent iron (ZVI) powders and other Fe-based metallic glasses. The influence of temperature (294–328 K) on the degradation reaction rate was measured using ball-milled metallic glass powders revealing a low thermal activation energy barrier of 22.6 kJ/mol. The excellent properties are mainly attributed to the heterogeneous structure consisting of local Fe-rich and Fe-poor atomic clusters, rather than the large specific surface and strong residual stress in the powders. The metallic glass powders can sustain almost unchanged degradation efficiency after 13 cycles at room temperature, while a drop in degradation efficiency with further cycles is attributed to visible surface oxidation. Triple quadrupole mass spectrometry analysis conducted during the reaction was used to elucidate the underlying degradation mechanism. The present findings may provide a new, highly efficient and low cost commercial method for azo dye wastewater treatment.

Azo dyes represent about one-half of the dyes used in the textile industry. It has been reported that about 20% of dyestuff is discharged directly into the environment by textile factories^1^,^2^. However, azo dyes are very difficult to decompose and often result in the pollution of water and irrevocable environmental problems^3^. New and effective treatment methods are needed for degrading azo dyes in order to reduce, or preferably eliminate, the amount of toxic and/or carcinogenic azo dye in wastewater. Traditional azo dye wastewater treatment methods usually filter or separate the organic chemicals through physical methods rather than decomposing the azo dye^4^. However, these methods cannot sufficiently eliminate azo dyes and their environmental impacts. Furthermore, the chemical and biological methods currently available are often more expensive and display even lower efficiency^5^,^6^, so their applications are largely restricted. The removal of pollutants in water by means of a reduction reaction using zero-valent metals has been developed recently as a new treatment technology. The most commonly used metal is zero-valent iron, which exhibits low cost, simple operation, and high efficiency^7^,^8^,^9^. However, ZVI is easily oxidized (rust) and its degradation efficiency decays rapidly. Recently, Fe-based amorphous powders and metallic glass composites have been developed that demonstrate higher efficiency in the degradation of azo dyes and other organic contaminants compared to conventional iron powders^10^,^11^,^12^. Metallic glasses have attracted considerable attention over the past three decades due to their unique physical, mechanical and chemical properties^13^,^14^. In particular, their good chemical and catalytic properties have been highlighted due to the far-from-equilibrium structures and high-energy states of the metallic glasses^15^,^16^,^17^,^18^. A long-range disordered atomic configuration gives metallic glasses a complex atomic and electronic structure, which is distinct from their crystalline counterparts and can contribute to excellent chemical and catalytic properties^18^. A shortcoming for using metallic glasses in applications requiring components with large physical dimensions are the size restrictions imposed by the glass formation ability^13^. However, for functional applications, such as dye degradation, powder samples can be prepared easily and there is little concern about any processing restrictions.

While there has also been interest in using Mg-based metallic glass and metallic glass composite powders for degrading azo dyes^19^,^20^,^21^, Fe-based metallic glasses represent a lower cost alternative and are thus most attractive for commercialization. Furthermore, in comparison to commercially pure ZVI, Fe-based metallic glasses generally contain some non-metallic elements, such as B and Si, and they have the potential to be tuned through micro-alloying to obtain the desired glass structure and properties. Although some Fe-based metallic glasses have been found to have high efficiency in degrading organic chemicals^10^,^11^,^12^, many issues are still poorly understood and require further investigation. This includes understanding the underlying mechanism of the degradation process, how it is influenced by the degradation environment and by the atomic and electronic configurations of the glass structure, and finally how to develop optimized Fe-based glass systems with higher degradation efficiency and service life.

Accordingly, in this paper we report a new yttrium containing Fe-based metallic glass powder (hereafter, denoted as G-ZVI) which has a much higher azo dye degradation efficiency compared to both a commercial crystalline ZVI powder (C-ZVI) and previously reported metallic glass powders^10^. Additionally, the effects of 1) annealing the G-ZVI powder, 2) changing the glass form (powder versus ribbon), and 3) changing the degrading environment were also studied to elucidate the possible degradation mechanism.

## Results

**Figure 1:**
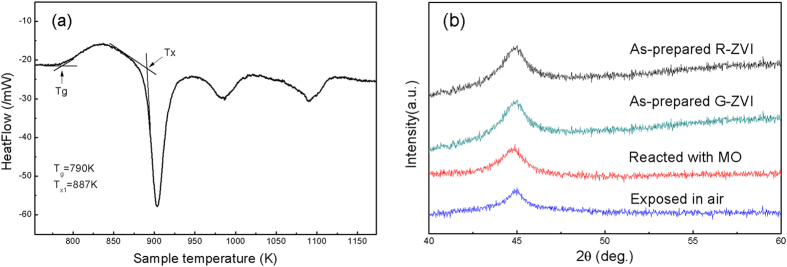
(**a**) The DSC trace of G-ZVI powder. (**b**) The XRD patterns of as-prepared ribbon (R-ZVI) and the ball milled G-ZVI powder, Also shown are patterns for G-ZVI reacted with methyl orange dye and G-ZVI exposed to air for one month, respectively.

**Figure 2:**
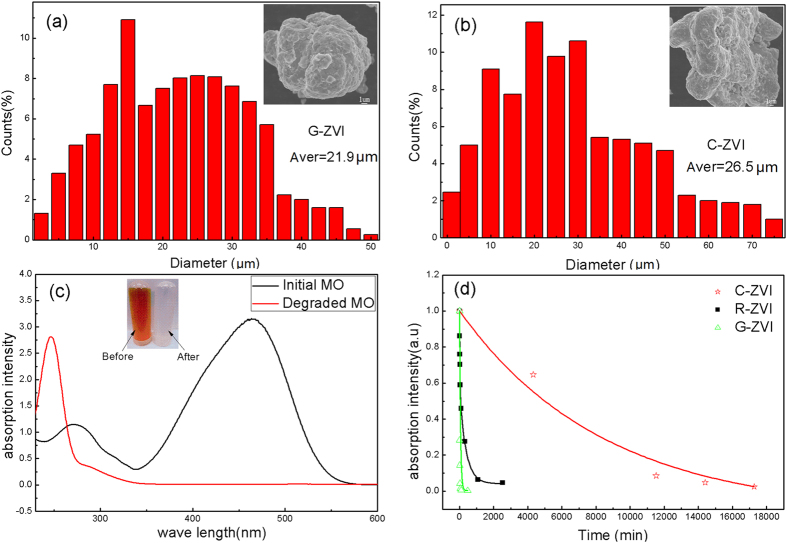
The size distribution of G-ZVI (**a**) and C-ZVI (**b**) powders after ball milling, inset with the corresponding particle morphologies. (**c**) The comparison of UV absorption spectra for as-prepared and completely degraded MO solution, inset with the appearance and color change before and after the reaction. (**d**) The normalized peak intensity at 464 nm as function of treatment time for G-ZVI, R-ZVI and C-ZVI for pH of 6 at a temperature of 294 K.

The DSC curve for G-ZVI powder is shown in Fig. 1a. The curve exhibits a characteristic endothermic peak for the glass transition and a super-cooled liquid region followed by three characteristic exothermic peaks for the sequential crystallization events. The glass transition temperature, T_g_, was found to be T_g_ = 790 K and the onset crystallization temperature, T_x1_, defined as the beginning point of the first crystallization event, was found to be T_x1_ = 887 K. XRD results show the amorphous nature of the as prepared amorphous ribbons (denoted as R-ZVI) and the G-ZVI powders ball-milled from R-ZVI (Fig. 1b). The G-ZVI powders show the same amorphous structure after methyl orange (MO) dye degradation experiments or exposure to air for 1 month (Fig. 1b), indicating their excellent stability. The size distribution of the G-ZVI and C-ZVI powders are presented in Fig. 2a,b, respectively. The results show that both types of powders have a narrow particle size distribution ranging from a few to tens of micrometers and the average diameters for G-ZVI and C-ZVI are 21.9 and 26.5 μm, respectively. Also, the similar surface morphology for both powders can be clearly seen after ball-milling (insets in Fig. 2a,b). Fig. 2c shows the UV absorption spectra for MO solutions before and after treatment, while the inset shows the appearance of the MO solutions. Only the results for G-ZVI reactant are shown in Fig. 2c since the same results were observed for the C-ZVI and R-ZVI reactants. From the UV absorption spectra, we can see two characteristic absorption peaks located at 464 nm and 271 nm, arising from the “–N = N–” azo bonding and benzene ring of MO, respectively. It is the absorption peak at 464 nm that makes MO appear orange. The absorption peak at 464 nm disappears and no obvious peak shift can be detected after the degradation treatment, while the absorption peak of the benzene ring shifts to 246 nm.

Although all three kinds of ZVIs are effective in decomposing the –N = N– bonding in MO, their degradation efficiencies are significantly different. The normalized UV absorption peak intensity at 464 nm, as a function of treatment time for the different ZVIs, is shown in Fig. 2d for the as-prepared MO solution at a temperature of 294 K. As can be seen, all of the curves indicate a decreasing intensity of the absorption peak at 464 nm, characteristic of the continuous degradation of MO. Here we use the time when the degradation ratio was 0.5, or half-life, to measure the degradation efficiency. The half-lives were ~5 min, 90 min and 5000 min for G-ZVI, R-ZVI and C-ZVI, respectively.

**Figure 3:**
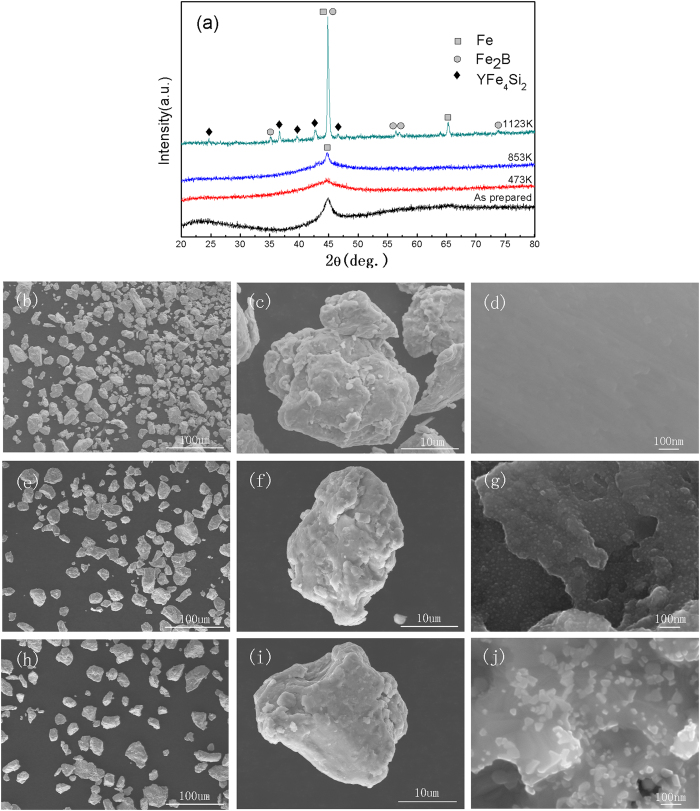
The XRD patters of the G-ZVI powder after 473 K, 583 K and 1123 K annealing, respectively (**a**) and the morphologies of the G-ZVI powder under different SEM magnifications after the 473 K (**b–d**), 853 K (**e–g**) and 1123 K (**h–j**) annealing, respectively.

The XRD patterns of the powders after annealing to induce structural relaxation are shown in Fig. 3a. The powder after annealing at 473 K shows an amorphous nature and the sample after annealing at 853 K has small amounts of crystalline Fe. Upon annealing at 1123 K, the amorphous powder has been completely crystallized into crystalline Fe, Fe_2_B and YFe_4_Si_2_, which is consistent with the DSC results (Fig. 1a).

Figure 3b–j show the powder size distribution, micrometer-scale and nanometer-scale morphologies of the annealed powders. As can be seen, all the particles are well dispersed with no observed aggregation and the size of all three types of particles ranges from a few micrometers to tens of micrometers, which is in agreement with the results shown in Fig. 2a. The particles have an irregular shape with roughness on their surface, which indicates the damage from ball-milling. However, there is a different appearance observed on the nanoscale of the surfaces. The surfaces of the powders after annealing at 473 K are clean, while those annealed at 853 K were interspersed with small amounts of white particles and those annealed at 1123 K were densely scattered with a large number of white particles with a particle size of tens of nanometers.

**Figure 4:**
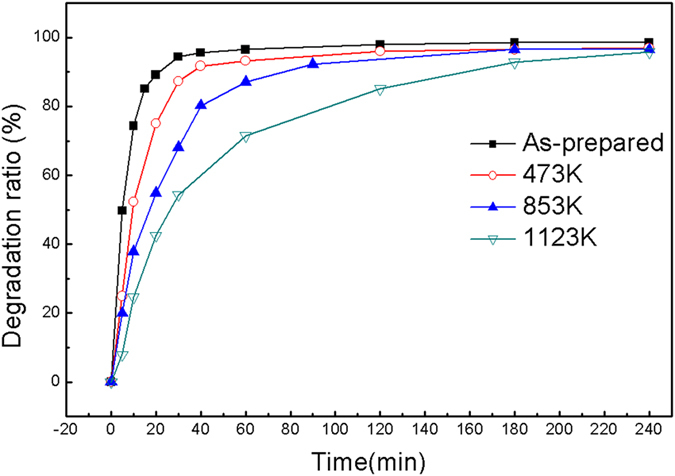
Plots of the degradation ratio versus treatment time for the degradation of MO by the G-ZVI powder in the as-prepared state and the G-ZVI powder annealed for one hour at 473, 853, and 1123 K.

Figure 4 shows the degradation ratio versus treatment time for G-ZVI after the different annealing treatments. It can be seen that the degradation efficiency for the different annealed G-ZVI samples differs greatly. Half-lives were ~5, 10, 20 and 30 min for the as-prepared G-ZVI and G-ZVI annealed at 473, 853 and 1123 K, respectively. It takes about 30, 60, 180 and 240 min to completely decompose MO in the solutions for the as-prepared G-ZVI and G-ZVI annealed at 473, 853 and 1123 K, respectively.

**Figure 5:**
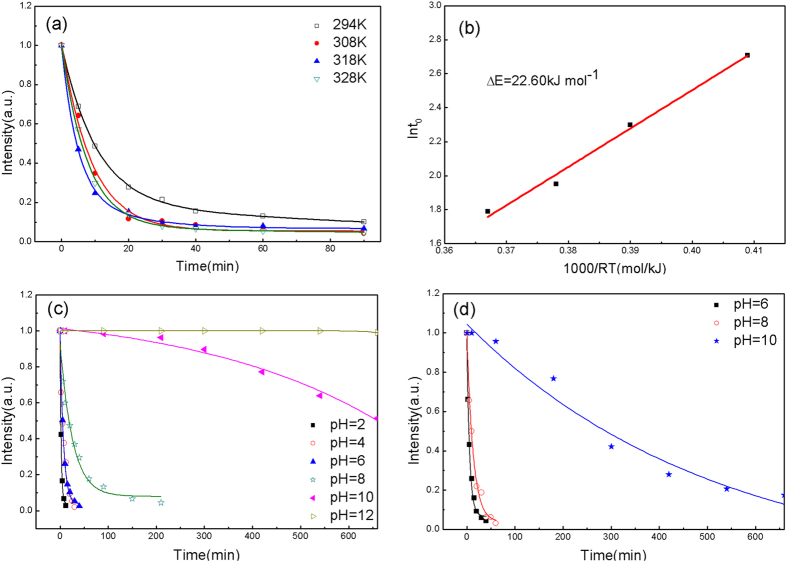
(**a**) The normalized peak intensity at 464 nm for MO as function of treatment time at different temperatures. (**b**) The plots of the decay time (t_0_) versus temperature for G-ZVI powders. The solid lines are the fitting by Eq. 2 to yield the activation energy. (**c**) The normalized peak intensity at 464 nm for MO as function of treatment time at different pH values (pH = 2, 4, 6, 8, 10, 12). (**d**) The normalized peak intensity at 464 nm for Congo red as function of treatment time at different pH values (pH = 6, 8, 10).

Figure 5a shows the relationship between the intensity of the UV absorption spectra and the treatment time at different temperatures from 294–328 K for as-prepared G-ZVI. With an increase in testing temperature, the degradation efficiency increases gradually. The degradation traces can be well fitted by an exponential decay function of a quasi-first order kinetic model:


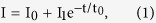


where I is the normalized intensity of the absorption peak at 464 nm, I_0_ and I_1_ are fitting constants, t is the treatment time and t_0_ is the time when the intensity decreases to e^−1^ of the initial state and can be derived by fitting the data points. We evaluated the thermal activation energy barrier (ΔE) with the Arrhenius-type equation,


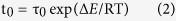


where R is the gas constant, T is the testing temperature and τ_0_ is a time pre-factor. The Arrhenius plots of ln(t_0_) vs 1/T are shown in Fig. 5b and the calculated ΔE value was 22.6 kJ/mol.

Figure 5c shows the relationship between the degradation ratio and the treatment time of G-ZVI for different pH solutions, indicating significantly different reaction efficiencies. The MO can be completely degraded very quickly under a low pH value environment and the reaction was rapidly decelerated upon increasing the pH value to a neutral or alkaline environment. No visible decolorization was observed after 24 hours for the MO solution with pH = 12, indicating a highly decelerated degrading efficiency in an extreme alkaline environment. Similarly, Fig. 5d shows the degradation rate of Congo red is improved for acidic pH values and slowed at alkaline pH value.

**Figure 6:**
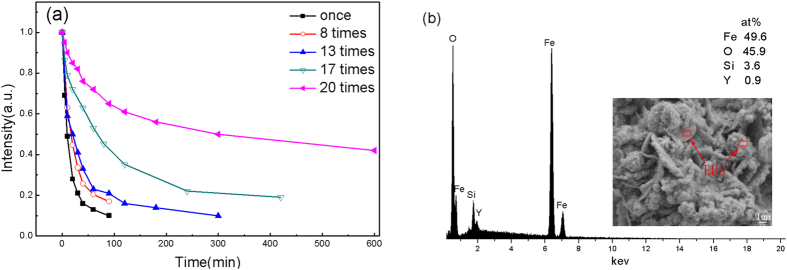
(**a**) The normalized peak intensity at 464 nm versus degradation time for different use cycles at room temperature. (**b**) The composition on the surface of the G-ZVI powder after experiencing 20 degradation cycles, inset shows the corresponding surface morphology.

For the service life evaluation of G-ZVI, Fig. 6a shows the degradation efficiency of the different cycles (up to 20 cycles). As can be seen, the degradation efficiency remains stable with no obvious change after 13 degradation cycles and then gradually declines, indicating a long service life for G-ZVI.

The EDS results shown in Fig. 6b indicate that the reaction products on the surface were mainly composed of Fe and O, indicating that the G-ZVI powder was significantly oxidized. A certain amount of Si and Y were also detected on the surface. B was not observed because it is beyond the detection range of EDS. The surface morphology of the G-ZVI powder that has experienced 20 degradation cycles is shown in the inset of Fig. 6b. The surfaces of the particles are rough and covered with abundant reaction products.

**Figure 7:**
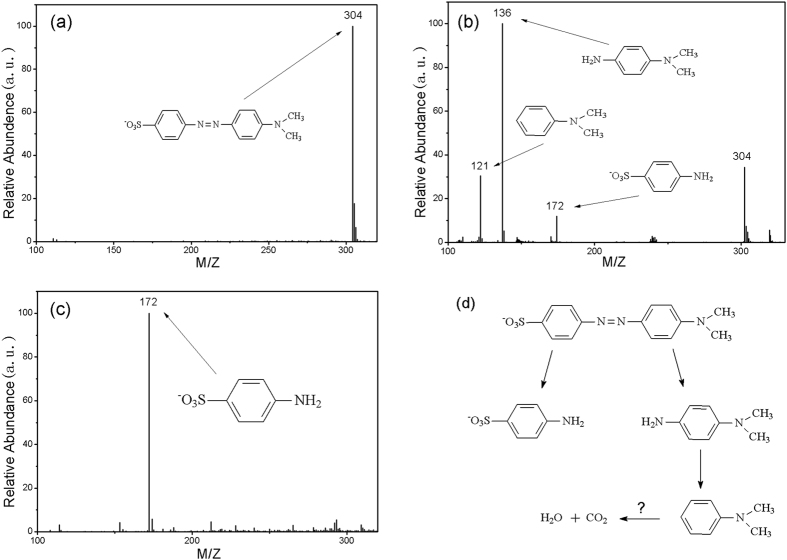
Full-scan product ion spectra corresponding to (**a**) the as-prepared MO solution, (**b**) the solution after 10 minutes of treatment, and (**c**) after 24 hours of treatement. Each peak is characterized by its m/z value. (**d**) A possible degradation pathway of MO.

Finally, Fig. 7a shows the total mass spectrogram for the as-prepared MO solution, on which there is an m/z peak at 304, which is characteristic of the ionization of MO. The mass spectrograms for the solutions after 10 min and 24 hours of degradation are shown in Fig. 7b,c, respectively. At the initial reaction stage (10 min), the m/z peak at 304 decreases by about 60% of the original intensity and three new m/z peaks at 121, 136 and 172 appear to represent the intermediate products of N,N-dimethylbenzenamine (C_8_H_11_N), N,N-dimethyl-p-phenylenediamine (C_8_H_12_N_2_) and sulfanilic acid (C_6_H_6_NSO_3_), respectively, indicating an effective degradation of MO. For the completely degraded solution (24 hours), the m/z peak at 304 disappears completely and only the m/z peak at 172 remains (Fig. 7c).

## Discussion

### Degradation efficiency of different ZVI samples

The disappearance of the 464 nm absorption peak, and the shift of the 271 nm absorption peak to 246 nm (Fig. 2c), show that the –N = N– bonding is completely decomposed, corresponding to the disappearance of orange color in the solution, and a new benzene ring is formed with a smaller molecular weight as a reaction product after the degradation reaction. Furthermore, in this study it is apparent that G-ZVI exhibits the shortest degradation time and highest degradation efficiency. Indeed, the half-life for the Fe_76_B_12_Si_9_Y_3_ G-ZVI is 5 min, which is roughly 18 and 1000 times faster than that for R-ZVI and C-ZVI, respectively.

Recognizing that there are significant differences in the raw material forms, the degradation experiment procedures, and the dyes used in various literature studies, it is useful to make some comparisons. The thermal activation energy barrier for the Fe_76_B_12_Si_9_Y_3_ G-ZVI in this study is 22.6 kJ/mol, which is somewhat smaller than published results for Fe_84_B_16_ (25.43 kJ/mol) and Fe_78_Si_8_B_14_ (27.9 kJ/mol) metallic glasses^22^,^23^. Moreover, the activation energy is far smaller than that for a Fe_73_Si_7_B_17_Nb_3_ (78 kJ/mol) metallic glass^10^. Finally, other researchers have found that Fe_78_Si_9_B_13_ metallic glasses only have 25 times the reaction efficiency of C-ZVI in degrading azo dyes^24^. Taking all above facts into consideration, we can conclude that G-ZVI we prepared has better degrading reactivity when compared to other amorphous Fe-based alloys.

The most direct comparisons can be made to the study by Wang *et al.*^10^ for Fe_73_Nb_3_Si_7_B_17_ since they used similar power preparation procedures and similar degradation experiments. That study found that ball-milled Fe_73_Nb_3_Si_7_B_17_ metallic glass powder has a half-life of 10 min for degrading –N = N– bonding, which is 60 times faster than that found for gas atomized metallic glass powder and 200 times faster than that found for commercial Fe powders with comparative particle size. By comparing to the study by Wang *et al.* it is found that the present Fe_76_B_12_Si_9_Y_3_ G-ZVI has a roughly two times higher efficiency than that of the previously reported Fe_73_Nb_3_Si_7_B_17_ metallic glass powder.

While Wang *et al.*^10^ attributed this excellent degradation property to the large specific surface area and strong residual stresses for the ball-milled samples, this does not fully explain the results in Fig. 2d for the present study. First, Wang *et al.* used a smaller powder particle size (8.6 μm) than the present study (21.9 μm), and thus had a higher specific surface area. Thus, the improved efficiency of the present G-ZVI can be attributed to compositional and/or structural differences and may be even greater for finer powder size. Next, R-ZVI exhibits much higher degradation efficiency than that found for C-ZVI, even though it has a much smaller specific surface area. Furthermore, the C-ZVI and G-ZVI powders have similar specific surface areas and residual stresses to due to the similar ball-milling procedure used, similar particle sizes and similar surface morphologies (Fig. 2a,b). However, C-ZVI has a 1000 times slower reaction efficiency when compared to G-ZVI. Thus, while the results of Wang *et al.* showed that the surface area and residual stress are important factors^10^, they are not the main factors for explaining the enhanced degradation efficiency of G-ZVI and R-ZVI relative to C-ZVI in the present study. While the present results confirm the importance of high surface area and residual stress from ball milling (G-ZVI vs. R-ZVI in Fig. 2d), they also suggest a much stronger effect from the amorphous versus crystalline structure (G-ZVI vs. C-ZVI in Fig. 2d).

### Effects of structural relaxation and crystallization

In order to confirm the importance of the amorphous structure, decomposition experiments for G-ZVI powders experiencing different degrees of structural relaxation and crystallization were performed. Based on the DSC results, three vacuum annealing temperatures were selected as T_1_ = 473 K (T_1_ ≪ T_g_), T_2_ = 853 K (T_g_ < T_2_ < T_x1_) and T_3_ = 1123 K (T_3_ ≫T_x1_). The data in Fig. 4 show that the degradation efficiency was the highest for the as-prepared G-ZVI, and decreases slightly for G-ZVI after low temperature relaxation at 473 K. At 853 K severe structural relaxation occurs with the onset of crystallization and the degradation efficiency deteriorates rapidly. Finally, the fully crystalized samples demonstrate the lowest degradation efficiency. Based on these results, it is reasonable to suggest an amorphous structure is definitely favorable for the degradation of MO and crystallization is detrimental to it. These results again confirm the importance of a disordered structure independent of the specific surface area; however, some questions arise. 1) What are the favorable traits of the amorphous structure that facilitate the degradation process, and how can we optimize it? 2) What is the underlying mechanism of the degradation of MO by G-ZVI and is it different to that by crystalline ZVI? Some clues may be found from the fact that a 1 hour vacuum annealing treatment far below the T_g_ begins to deteriorate the degradation efficiency. As is well accepted, low temperature annealing changes the chemical short range ordering (CSRO) in metallic glasses^25^. For relatively low T_g_ BMGs, distinct changes in the CSRO at very small length scales can be observed even at room temperature^26^,^27^. For an (Fe_71.2_B_24_Y_4.8_)_96_Nb_4_ Fe-based metallic glass, Mössbauer spectra have revealed a local atomic structure with an inhomogeneous distribution of Fe atoms, with localized Fe-rich regions and Fe-poor regions, which can be adjusted by annealing below T_g_^28^. A similar Mössbauer spectroscopy analysis was not possible for the present metallic glass composition since it is not ferromagnetic. However, it is quite reasonable to speculate that an inhomogeneous distribution of Fe-rich or Fe-poor clusters occurs in the present Fe-based metallic glass as well, which can be distinctly adjusted by low temperature structural relaxation. The optimal Fe-rich clusters act in the degradation process and the metalloid elements (Si, B) and rare earth element (Y) help to form these amorphous Fe-rich clusters. The efficiency drops with a decrease in the number of the favorable Fe-rich clusters after low temperature structural relaxation and continues to drop continuously with the onset of crystallization, indicated as Fig. 4. The importance of the inhomogeneous glassy structure can also explain why the as-prepared R-ZVI, which contains the same favorable Fe-rich amorphous clusters that G-ZVI possess, also has relatively high efficiency (~100 times C-ZVI) for degrading MO in spite of the much lower surface area.

As is well known, a typical redox reaction is responsible for the ZVI degradation of azo dyes^29^,^30^. So the ability of the reducing agent to lose electrons partly determines the degradation efficiency. When compared with the Fe-rich nano-clusters, the Fe-poor regions mainly coordinate with the metalloid elements and exhibit a relatively low electronegativity. So it is easy to envision galvanic cells between them, which promote the Fe atoms in the Fe-rich clusters to lose electrons and take part in the degradation reaction. Generally, the local atomic Fe-rich and Fe-poor clusters are in the nanometer scale and are assumed to be homogeneously distributed in the metallic glass. So a large quantity of nano-galvanic cells is expected with a strong affinity for donating electrons to the reaction. However, understanding the mechanistic details of the galvanic cells, and how to improve their performance, will require further investigation.

### The influence of the degrading environment

The thermal activation energy barrier, ΔE, of 22.6 kJ/mol found for the Fe_76_B_12_Si_9_Y_3_ G-ZVI in the present study is much lower than that previously found for ball-milled Fe_73_Nb_3_Si_7_B_17_ metallic glass powder (78 kJ/mol)^10^. This result is consistent with the improved degradation efficiency that was found for Fe_76_B_12_Si_9_Y_3_. Both G-ZVI powders were prepared using ball-milling and display similar particle size and surface morphology, with only a slight difference in their composition. Most notably, the Fe-based metallic glass adopted here has 3% Y, while Wang *et al.* used a composition with 3% Nb^10^. It is well accepted that micro-alloying metallic glasses with rare earth elements can regulate the glass structure and promote improved properties such as high glass forming ability, high toughness and high resistance to corrosion^31^,^32^. The results here show that rare earth element micro-alloying can significantly improve the azo dye degrading ability, which is likely related to the alloying additions promoting favorable Fe-rich clusters in the G-ZVI glass structure. So, the combined results of these studies provide clues to aid in the pursuit of metallic glass compositions optimized for decomposing organic chemicals.

However, both the degrading experiments for MO and Congo red dye (as shown in Fig. 5d) indicate that it is the nature of G-ZVI to only efficiently decompose organic azo dyes under strong acidic or near neutral conditions. For commercial ZVI it is well known that iron reacts with H_2_O to form ferrous hydroxide or ferric hydroxide, which is insoluble in water and covers the ZVI surface to block the degradation reaction^33^,^34^. In the case of G-ZVI, it is found that an Fe-based metallic glass with 24 at. % non-iron elements can also similarly react with H_2_O to form a detrimental hydroxide surface film.

### Degradation products and pathway

As described above, Fe atoms are active in the degradation reaction; however, Fe can be easily oxidized covering the surfaces with iron oxides. This separates ZVI from direct contact with the azo dyes and thus impedes further degradation reaction. Also, red ferrous oxides visible to the naked-eye can be observed on the surface of metallic glass powders after 20 degradation cycles. Thus, the loss in degradation efficiency with repeated use (Fig. 6a) is attributed to surface oxidation that slows further degradation reaction. However, it should be noted that amorphous G-ZVI has a relatively good oxidation resistance and can maintain almost unchanged degradation efficiency after 13 cycles, which is much better than amorphous Fe_84_B_16_, (Fe_0.99_Mo_0.01_)_78_Si_9_B_13_ and Fe_78_Si_9_B_13_ ribbons, which can only be reused no more than 4 times with high efficiency^23^,^35^,^36^. The excellent structural stability is attributed to the large amount of non-metallic elements, which impede the formation of an oxide film on the particles^22^.

In order to understand the reaction process, mass spectrometry analysis was applied to detect the intermediate products during the degradation process. The results (Fig. 7) show that the intermediate products are of N,N-dimethylbenzenamine (C_8_H_11_N), N,N-dimethyl-p-phenylenediamine (C_8_H_12_N_2_) and sulfanilic acid (C_6_H_6_NSO_3_), and that they are further degraded during the degradation experiments. After completion, the MO was decomposed into sulfanilic acid and other small molecule organics or even into carbon dioxide and water, which are beyond the detection range of mass spectrometry. According to this scenario, a degradation mechanism for MO by G-ZVI is suggested (as shown in Fig. 7d).

## Conclusion

A new Fe-based metallic glass (Fe_76_B_12_Si_9_Y_3_) with extraordinary ability to efficiently degrade MO in strong acidic or near neutral environments has been reported in this paper. The efficiency is up to 1000 times of crystalline iron powder and approximately 2 times of a previously reported Fe-based metallic glass produced and tested under similar conditions. The thermal activation energy barrier for the degradation reaction was very low (22.6 kJ/mol), which also confirmed its quick reaction rate. Results suggest the favorable properties are mainly attributed to the amorphous structure of local Fe-rich and Fe-poor atomic clusters, while the large specific surface area or strong residual stress of the ball-milled powders contributes a smaller effect. It is proposed that the nanometer Fe-rich clusters and Fe-poor regions rich in metalloid elements may provide numerous nano-galvanic cells to help Fe to lose electrons and therefore, decompose organic chemicals. The favorable amorphous structure can be degraded or destroyed after low temperature structural relaxation or crystallization, respectively, corresponding to a modest or large drop in the degradation efficiency, respectively. At room temperature, the G-ZVI powders have good structural stability in air and can sustain almost unchanged degradation efficiency after 13 cycles under these experimental conditions. The drop in degradation efficiency upon further cycles was attributed to the surface oxidation of the material, which created a barrier that reduced the contact of the G-ZVI with the azo dye. The underlying degradation mechanism of G-ZVI towards MO was discussed based on the intermediate products detected by mass spectrometry analysis during the reaction. These findings may provide a new highly-efficient and low cost commercial method for azo dye wastewater treatment.

## Methods

### Materials

A G-ZVI with a nominal composition of Fe_76_B_12_Si_9_Y_3_ (at. %) was selected for this study. Ribbon samples (R-ZVI) were produced by melt-spinning the alloy melts at 36 m/s (VF-RQT50, MAKABE R&D Co. LTD., Japan) and the G-ZVI powder was prepared from R-ZVI by high energy ball-milling (QM-3SP2, Nanjing University instrument plant, China) the ribbons under a high purity argon atmosphere for ~3000 min at 300 rpm. The ball-miller rotated and then paused using alternating 6 min time intervals to avoid an increase in temperature. The purchased C-ZVI (Tianjin Yongda Chemical Reagent Co. Ltd.) had an original particle size of 43.7 μm and >98% purity. In order to obtain comparative particle sizes and surface morphology, the C-ZVI was also prepared via ball-milling with the same procedures, but a shorter time of 1200 min was needed since the material started in powder form. The particle size and distribution of the ball-milled Fe powders were inspected using a laser particle analyzer (BT9300ST, Dandong Bettersize Technology Co., Ltd., China). The crystallization temperature (T_x_) and glass transition temperature (T_g_) of the G-ZVI powders were studied using differential scanning calorimetry (DSC, Setaram 16–18, France) with a constant heating rate of 20 K/min. To study the effect of relaxation and crystallization, G-ZVI powders were heated using a constant heating rate of 20 K/min in a vacuum oven (~5*10^−3^ Pa) followed by annealing at 474 K, 853 K, or 1123 K for 1 hour before allowing to cool in the switched off furnace. The crystallization behavior and phase constitution of the R-ZVI ribbon and G-ZVI powders were characterized using X-ray diffraction (XRD) with Cu-Kα radiation at a scan rate of 12 degree per min (D8-advance, BRUKER, Germany). The surface morphology of the G-ZVI and C-ZVI samples after ball-milling and G-ZVI powders after structural relaxation were investigated using scanning electron microscopy (SEM, SU-70, HITACHI, Japan) with energy dispersive spectroscopy (EDS).

### Dye Degradation Experiments

For all the degradation experiments, 40 mg of the metallic degradation reagent was added to 10 mL methyl orange dye (MO, C_14_H_14_N_3_SO_3_) solution with an initial MO concentration of 20 mg/L. The degradation process was determined by the strength of the maximum absorbance wavelength of MO in the ultraviolet absorption spectrum (UV-2450 spectrometer, Shimadzu, Japan). The degradation ratio (D) was calculated using the following equation:


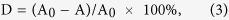


where A_o_ and A are the strength of the maximum absorbance wavelength of MO before and during the degradation process, respectively. The degradation products of MO were analyzed using triple quadrupole mass spectrometer (Bruker amaZon SL, Germany) with an electrospray ionization (ESI) source (Thermo Scientific, San Jose, USA). High purity nitrogen was used as nebulizer and auxiliary gas. The ESI probe tip and capillary potentials were set at 4.5 kV and −8 V, respectively. The selected reaction monitoring (SRM) method was selected to monitor the precursor-to-product ion transitions of m/z 50 to 800 at negative ionization mode.

Next, to further understand the degradation mechanism of G-ZVI towards MO, the UV absorption spectra of the solutions were measured at various temperatures (294, 308, 318, 328 K) and for various pH values (pH = 2, 4, 6, 8, 10, 12) at ambient temperature (294 K). The pH of the as-prepared MO solution is 6 and it was adjusted to lower or higher values by adding 0.1 mol/L HCl solution or 0.1 mol/L NaOH solution, respectively. The pH values were tested using a high precision pH meter (PHB-3, Shanghai DM Optical Instruments Co. LTD. China). Since MO exhibits different molecular structures in acidic versus alkaline solutions, the same experimental procedure was used to degrade Congo red (C_32_H_22_N_6_Na_2_O_6_S_2_), another azo dye that exhibits the same structure at different pH levels over the range of pH = 6, 8, 10.

Finally, to study the cycle life of G-ZVI, the following experimental procedures were used. First, 40 mg of G-ZVI powder was added to 10 mL of MO solution with a concentration of 20 mg/L. Two days later, after the complete decomposition of MO, the solution was removed and replaced by the same amount of fresh MO solution. This process was repeated until the degradation efficiency dropped significantly. After degradation experiments, the structural and composition changes of G-ZVI were evaluated by XRD, SEM and EDS.

## Additional Information

**How to cite this article**: Xie, S. *et al.* A highly efficient degradation mechanism of methyl orange using Fe-based metallic glass powders. *Sci. Rep.* **6**, 21947; doi: 10.1038/srep21947 (2016).
